# Gender Differences of NLRP1 Inflammasome in Mouse Model of Alzheimer's Disease

**DOI:** 10.3389/fnagi.2020.512097

**Published:** 2020-11-19

**Authors:** Jinghang Zhang, Lihong Pei, Dan Zang, Yun Xue, Xiaohui Wang, Yiyang Chen, Jinsong Li, Jian Yu, Qingzu Gao, Wenyu Di, Chaochu Cui, Wei Su, Xianwei Wang

**Affiliations:** ^1^Department of Pathology, The First Affiliated Hospital of Xinxiang Medical University, Xinxiang, China; ^2^Key Laboratory of Lymphohematopoietic Tumor in Xinxiang, Xinxiang Medical University, Xinxiang, China; ^3^Henan Key Laboratory of Medical Tissue Regeneration, Xinxiang Medical University, Xinxiang, China

**Keywords:** Alzheimer's disease, NLRP1 inflammasome, gender differences, neuronal apoptosis, APP/PS1^+/−^ mice

## Abstract

**Background:** There is a significant gender difference in the incidence and symptoms of Alzheimer's disease (AD), but its mechanisms are not completely understood. Recent studies showed that NLRP1 inflammasome was overexpressed in females under some pathological conditions such as nodular melanoma. Whether NLRP1 signals have a gender difference in AD has not been elucidated. This study was designed to investigate gender difference on the expressions of NLRP1 signals including NLRP1, Capase-1 and IL-1β in the brains of APP/PS1^+/−^ mice.

**Methods:** Female and male APP/PS1^+/−^ mice (30-weeks-old) were used in this study. Amyloid-β (Aβ) plaques were stained with Congo red dye and cell apoptosis was detected by TUNEL staining. Expressions of NLRP1, Capase-1 and IL-1β were measured by immunofluorescent staining and Western blotting assay.

**Results:** The numbers of Aβ plaques in cortex and hippocampus and neuronal apoptosis in cortex were 4 and 2-folds in females than males, respectively (*P* < 0.001). The average size of Aβ plaques in both cortex (females: 3527.11 ± 539.88 μm^2^ vs. males: 1920.44 ± 638.49 μm^2^) and hippocampus (females: 1931 ± 308.61 μm^2^ vs. males: 1038.55 ± 220.40 μm^2^) were also larger in females than males (*P* < 0.01). More interestingly, expressions of NLRP1, Caspase-1, and IL-1β were markedly increased in the cortex of females as compared with males.

**Conclusions:** These findings show that NLRP1 signals are higher in brains of female APP/PS1^+/−^ mice than males, which may be related to the gender differences of AD.

## Introduction

Alzheimer's disease (AD) is a progressive neurodegenerative disorder that is characterized by symptoms like memory impairment, cognitive dysfunction, perception disturbance and language problems, and by neuropathological alterations including excess deposition of extracellular beta-amyloid peptides (Aβ), aggregation and accumulation of intracellular hyperphosphorylated tau protein, and neuronal loss in some areas of the brain (Dos Santos Picanco et al., 2018; Veitch et al., 2019). There are gender differences in the incidence of AD, approximate two-thirds of the diagnosed cases happen in women, and the symptoms and neuropathological alterations in female patients are more severe than males (Hebert et al., 2010; Filon et al., 2016; Canevelli et al., 2017). However, the reason for these gender differences in AD remains currently elusive.

Inflammasomes are multiple protein complexes that promote the cleavage of caspase-1 precursor proteins and the release of inflammatory factors such as IL-1β and IL-18 leading to inflammatory responses (Schroder and Tschopp, 2010; Liu et al., 2017). In the body, inflammasomes are recognized by microbial toxins, pathogen-associated molecular patterns (PAMP) and danger associated molecular patterns (DAMP) (Schroder and Tschopp, 2010). The recognition molecules of inflammasomes are classified into nucleotide-binding oligomerization domain-like receptors (NLR) and hemopoietic interferon-inducible nuclear receptors (HIN) (Schroder and Tschopp, 2010). NLRs are divided into several subclasses such as NLRP1, NLRP2, NLRP3, and NLRC4. HINs contain AIM2 inflammasome (Lamkanfi and Dixit, 2014; Shi et al., 2015). Recent studies have shown that activation of NLRP inflammasomes is relevant to the pathogenesis of cardio-cerebrovascular diseases (Singhal et al., 2014; van Hout and Bosch, 2018).

It has been demonstrated that NLRP1 and NLRP3 are expressed in neurons and microglia in the brain (Gustin et al., 2015; Tan et al., 2015), and mediate neuronal injury in neuropathological states such as ischemic stroke (Fann et al., 2013). A recent study showed that both NLRP3 and NLRP1 are activated in AD (Saresella et al., 2016). The inflammasome components including NLRP1, NLRP3, PYCARD, caspase-1,−5, and−8 and their downstream effectors IL-1β and IL-18 were markedly upregulated in mild and severe AD at transcriptional level (Saresella et al., 2016). Increasing evidence also indicates that the excessive activation of both NLRP1 and NLRP3 contributes to pathogenesis of AD (Heneka et al., 2013; Tan et al., 2014). Tan et al. showed that the activation of NLRP1/caspase-1 axis mediated the neuronal pyroptosis and promoted AD progression in the mouse AD model APP/PS1 (Tan et al., 2014). Heneka et al. reported that NLRP3/caspase-1 axis also participated in AD pathogenesis, and NLRP3 inflammasome activation contributed to the activation and deposition of amyloid-β in the APP/PS1 mice (Heneka et al., 2013).

Studies have indicated that both NLRP1 and NLRP3 inflammasomes are expressed and function in a gender-dependent manner in some pathological states (Verma et al., 2012; Wu et al., 2016). Recently, Verma et al. found that NLRP1 variant was significantly more common in fair-skinned female patients, however, NLRP3 variant was more common in male patients, which was associated the pathogenesis of nodular melanoma (Verma et al., 2012). This finding suggests that NLRP1 may play a more important role in some female high-risk diseases. Meanwhile, Tan et al. reported that NLRP1 was markedly increased in the brains of APP/PS1 transgenic mice (6-months-old and 9-months-old males) as compared with the aged- and gender-matched non-transgenic mice, but there was no difference between the 3-months-old transgenic and non-transgenic mice (Tan et al., 2014). This study suggests that NLRP1 expression would be increased in the old (≥6 months) APP/PS1 transgenic mice. However, there is still not any literature to compare the difference of NRLP1 between male and female APP/PS1 transgenic mice. This study was designed to investigate the expression and activity of NLRP1 signaling in the cerebral cortex of APP/PS1^+/−^ mice (30-weeks-old) of different sexes.

## Materials and Methods

### Animals

APP/PS1^+/−^ mice were purchased from the Model Animals Research Center of Nanjing University [license number: SCXK (Su) 2010-0001], and APP/PS1^+/−^ male mice and wild type (WT) C57BL/6 female mice were used for cage breeding. The phenotype of APP/PS1^+/−^ mice [B6. C3-Tg (APPswe, PSEN1dE9) 85Dbo/J] were identified by genotyping, and were used at 30 weeks of age (*n* = 40, 20 males and 20 females; Li et al., 2016; vonderEmbse et al., 2017; Agostini et al., 2020). Mice were kept in a 12:12 light-dark cycle at room temperature 22–25°C with relative humidity 60–80%, and free access to standard laboratory chow and drinking water. Animal protocols were approved by the Animal Care and Use Committee of Xinxiang Medical University and conformed the Guide for Care and Treatment of Experimental Animals published by the Ministry of Science and Technology of the People's Republic of China (Beijing, China).

### Hematoxylin & Eosin Staining Staining and Congo Red Staining

Mice were fixed by cardiac perfusion with 4% paraformaldehyde following anesthesia by an intraperitoneal injection of 5% chloral hydrate. Brains were collected and placed in 4% paraformaldehyde. The brain was divided into two hemispheres. Each hemisphere was cut into 5 μm thick slices with the coronal direction by cryostat (Leica, CM1950, Germany). Slices of brain were made and spread on slides coated with polylysine. Congo red staining was performed according to the instructions of the kit (G1530, Solarbio Science & Technology, Beijing, China). HE staining was performed as per the standard protocols. The images were taken with 20 × or 40 × amplification (Congo red or HE staining slices) with a fluorescence microscope (Olympus, BX61, Japan). Each image was analyzed using Image *J* (NIH) software.

### TUNEL Staining

Cell apoptosis was detected using DeadEnd^TM^ Fluorometric TUNEL Apoptosis Detection Kit (G3250, Promega Corporation, Fitchburg, WI, USA) as per the manufacturer's instructions. The images were acquired with a fluorescence microscope (Olympus, BX61, Japan). Each image was analyzed using Image *J* software (NIH). There were three random fields chosen in every image to be analyzed.

### Immunofluorescence Staining

The brain sections were treated with citrate buffer, blocked with 3% goat serum/1% BSA and incubated with primary antibodies (1:500 dilution) at 4°C overnight. After washing with PBS, the sections were incubated with fluorescent-labeled secondary antibodies for 2 h at room temperature in the dark. This was followed by washing with PBS three times and deionized water one time, then the sections were mounted with DAPI in antifade mounting medium (1:1000 dilution, S2100,Solarbio Science & Technology, Beijing, China), and the images were photographed by a fluorescence microscope (Olympus, BX61, Japan). Each image was analyzed using Image J software (NIH) (*n* = 3). The primary antibodies and secondary antibodies were as follows: rabbit polyclonal anti-NeuN (ABN78, Millipore, Billerica, USA), rabbit polyclonal anti-NLRP1 (sc-66993, Santa Cruz, CA, United States), rabbit polyclonal anti- IL-1β (ab9722, Abcam), rabbit polyoclonal anti-Caspase1 (22915-1-AP, Proteintech), Alexa Fluor Plus 488 goat anti-Rabbit secondary antibody (1:600 dilution, A32723, Thermo Fisher Scientific) and Alexa Fluor Plus 594 goat anti-Mouse secondary antibody (1:800 dilution, A32723, Thermo Fisher Scientific).

### Western-Blotting Assay

Proteins were extracted from mouse brains with RIPA Lysate buffer (P0013C, Beyotime, China). The protein concentrations were measured using the BCA method (23225, Thermo Fisher Scientific). The proteins (30 μg) were separated by electrophoresis with 10% SDS-PAGE gels, and then transferred onto PVDF membranes. The blots were blocked with 5% skim milk for 1 h at room temperature, and then incubated with primary antibodies (1:1000 dilution) at 4°C overnight. Primary antibodies included: rabbit polyclonal anti-NLRP1 (sc-66993, Santa Cruz, CA, United States), rabbit polyclonal anti-IL-1β (ab9722, Abcam), rabbit polyoclonal anti-Caspase1 (22915-1-AP, Proteintech, Wuhan, China). Following washing with TBST three times, the blots were incubated with HRP-labeled goat anti-rabbit secondary antibodies (1:2000 dilution, ZB-5301, Beijing Zhongshan Golden Bridge Biotechnology, Beijing, China) for 1 h at room temperature. Following washing with TBST, the blots were exposed to ECL luminescent solution for 3 min (RPN2232, ECL Plus Western Blotting Detection System, GE Healthcare). Strip analysis was performed by Quantity One 4.6 software (*n* = 3).

### Statistical Analysis

Each experiment was replicated 3–4 times with 3–4 mice per condition. Statistical analysis was performed using GraphPad Prism 6.0 software. The data were presented as means ± standard deviation (mean ± *SD*). Student *t*-test was used for comparison between two groups. Gaussian distribution analysis was conducted before the *t*-test. If the data did not conform to the Gaussian distribution, Welch's correction was conducted first. *P* < 0.05 was considered as statistical significance.

## Results

### Gender Differences of Aβ Plaques and Neuronal Apoptosis in Brains of APP/PS1^+/-^ Mice

**Figure 1:**
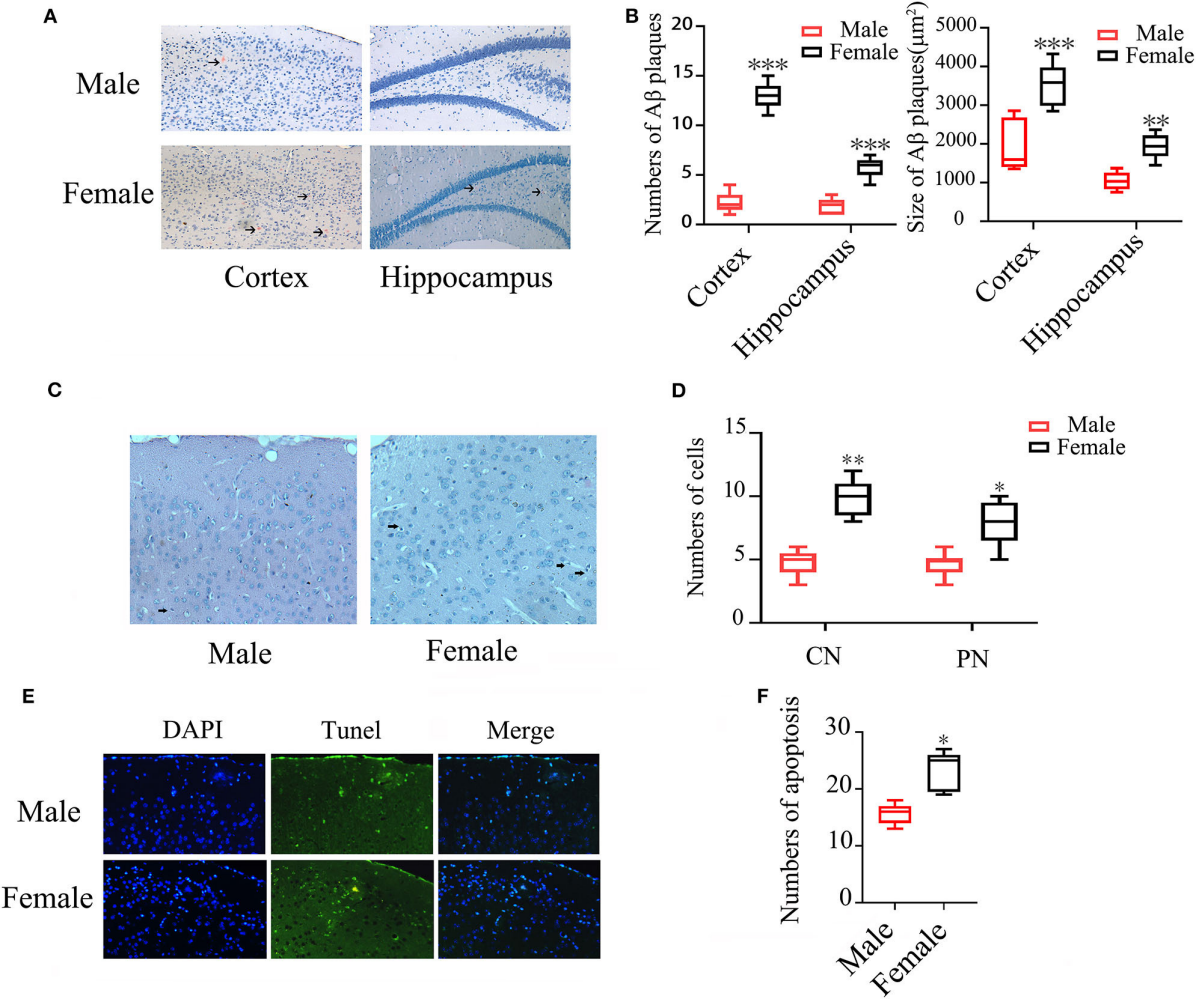
Aβ plaques and neuronal apoptosis in brains of male and female APP/PS1^+/−^ mice. **(A)** Congo red staining showing Aβ plaques in the cortex and hippocampus of APP/PS1^+/−^ mice; **(B)** Quantification of Aβ plaque numbers. **(C)** HE staining showing condensed (CN) and pycnotic nuclei (PN) (arrows indicated) in cortex of APP/PS1^+/−^ mice; **(D)** Quantification of the numbers of condensed and pycnotic nuclei; **(E)** TUNEL staining showing neuronal apoptosis in the cortex of APP/PS1^+/−^ mice; **(F)** Quantification of the numbers of apoptotic cells. *n* = 8–10. *P < 0.05, **P < 0.01, and ***P < 0.001 vs. male mice.

Congo red staining showed that the numbers of Aβ plaques were 4-folds higher in both cortex and hippocampus of female APP/PS1^+/−^ mice than the otherwise identical male mice (*P* < 0.001). The average size of Aβ plaques in both cortex (females: 3527.11 ± 539.88 μm^2^ vs. males: 1920.44 ± 638.49 μm^2^ and hippocampus (females: 1931 ± 308.61 μm^2^ vs. males: 1038.55 ± 220.40 μm^2^) were also larger in females than males (*P* < 0.001, *P* < 0.01) (Figures 1A,B). Hematoxylin/Eosin (HE) staining showed that the numbers of condensed and pycnotic nuclei in cortex of female mice were significantly higher than males (*P* < 0.01, *P* < 0.05) (Figures 1C,D), which indicates neuronal apoptosis. The neuronal apoptosis was further confirmed by TUNEL (Figure 1E), which also showed an increased 50.64% apoptotic cells in female APP/PS1^+/−^ mice (*P* < 0.05) (Figure 1F).

### Gender-Different Expression of NLRP1, IL-1β, and Caspase 1 in Cortex of APP/PS1^+/-^ Mice

**Figure 2:**
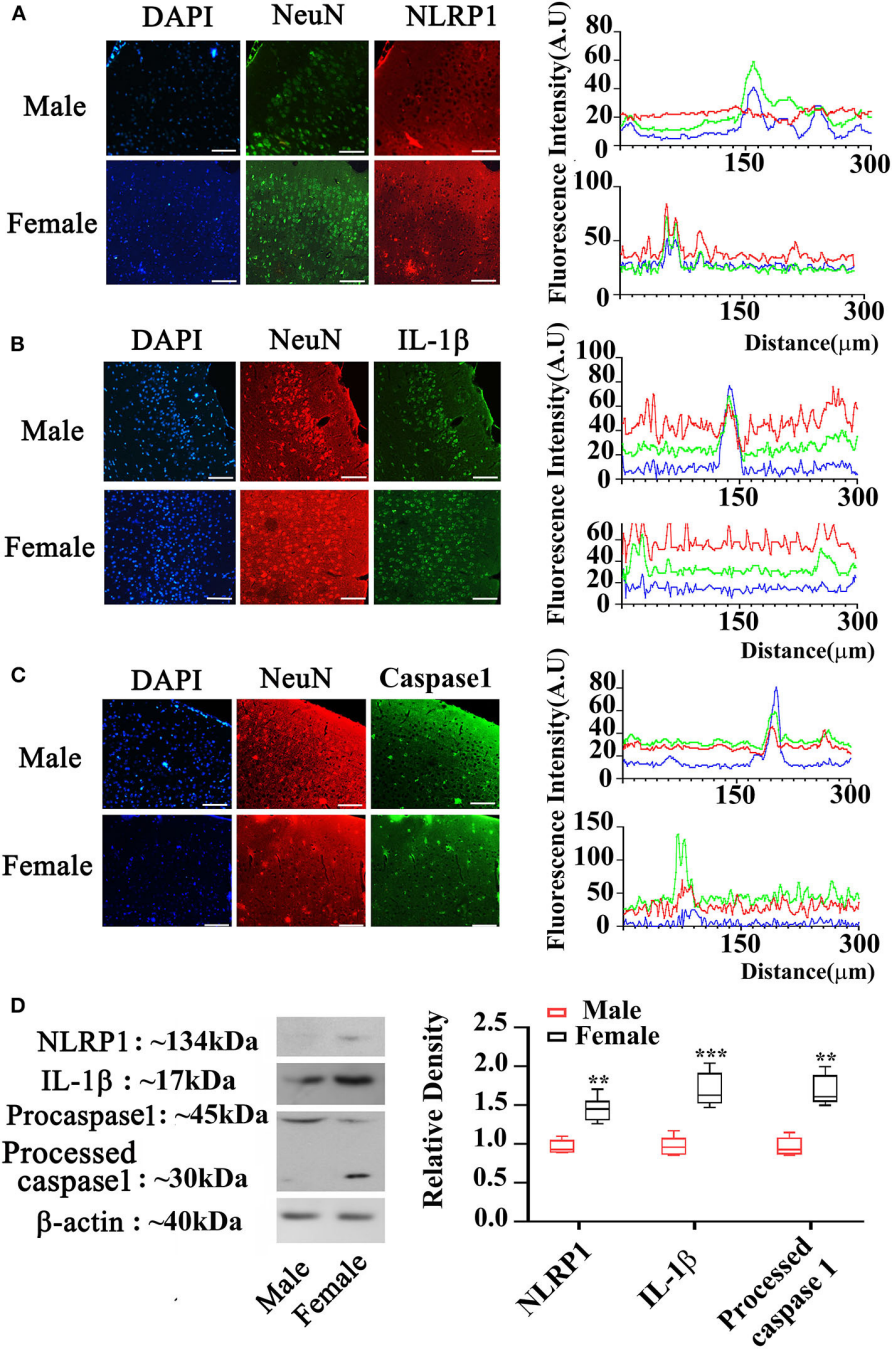
Expression of NLRP1 signaling in cortex of male and female APP/PS1^+/−^ mice. **(A–C)** Immunostaining showing NLRP1, IL-1β, and Caspase 1 expression in the cortex of male and female APP/PS1^+/−^ mice; and the blue, red and green fluorescence in the right panel are for DAPI, NeuN, and IL-1β, respectively; **(D)** Western-blotting staining showing NLRP1, IL-1β, and Caspase 1 expression in cortex of male and female APP/PS1^+/−^ mice (the line in each box in the right panel indicating means). **P < 0.01 and ***P < 0.001 vs. male mice. The sale bar = 50 μm in low magnification images. *n* = 8–10. The sale bar = 20 μm in insert images.

**Figure 3:**
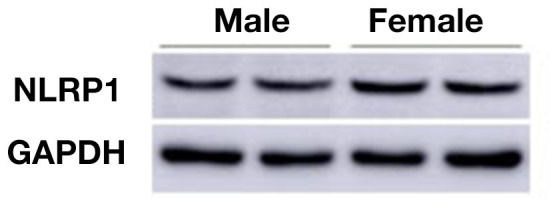
Western-blotting data shows NLRP1 expression in male and female WT mice (30-week-old).

To elucidate the expression of NLRP1 signaling, we measured molecular members of NLRP1 inflammasome including NLRP1, IL-1β, and Caspase 1 using immunohistochemistry. As shown in Figures 2A–C, the expression (fluorescent density) of NLRP1, IL-1β, and Caspase 1 in cortex of female APP/PS1^+/−^ mice was markedly higher than the male APP/PS1^+/−^ mice. This data was further confirmed by Western-blotting assay which also showed marked increases of NLRP1, IL-1β, and Caspase 1 in cortex of female APP/PS1^+/−^ mice (*P* < 0.01, *P* < 0.05) (Figure 2D). Of note, NLRP1 was also observed to be slightly upregulated in the brain of female WT mice as compared with the WT male mice (Figure 3).

## Discussion

Clinical studies have shown that women have a higher risk of suffering AD than men, and the clinical symptoms of female AD patients are more serious than males (Hebert et al., 2010; Filon et al., 2016; Canevelli et al., 2017). The mechanisms underlying the gender differences of AD are still not completely understood. In this study, we compared NLRP1 signaling in the cortex of male and female APP/PS1^+/−^ mice. We found that the expressions of NLRP1 inflammasome components including NLRP1, IL-1β, and Caspase 1 were all markedly increased in the cortex of female APP/PS1^+/−^ mice as compared with the males. In addition, we also observed marked increases of Aβ plaques in cortex and hippocampus and neuronal apoptosis in cortex of female APP/PS1^+/−^ mice as compared with males.

The activation of inflammasomes is one of the important mechanisms by which the body exerts innate immune responses (Evavold and Kagan, 2019). The abnormal activation of inflammasomes participates in the development and progression of multiple types of diseases. Inflammasomes include a NLRP1, NLRP2, NLRP3, AIM2, and NLRC4. Among them, NLRP1 and NLRP3 have been proved to play an important role in neuroinflammation and pathogenesis of AD (Saresella et al., 2016). Recently, Saresella et al. identified the expressions of 84 genes involved in the assembly of inflammasomes using qPCR assay and found that NLRP3 and Caspase 8 were markedly increased in AD patients (Saresella et al., 2016). Meanwhile, the downstream signals of Caspase 8, IL-1β, and IL-18 were also markedly increased in AD patients^23^. Following, pro-inflammatory cytokines IL-1β and IL-18. Yap et al. showed that the activation of NLRP1 inflammasome contributed to Aβ aggregation and neuroinflammation in the central nervous system (CNS) (Yap et al., 2019). The activation NLRP1 subsequently activates Caspase-1 and processes IL-1β and IL-18 into maturation, and promotes neuronal apoptosis and axonal degeneration (Yap et al., 2019). Knockout of NLRP1 and Caspase-1 could markedly inhibit neuroinflammation and axonal degeneration (Kaushal et al., 2015). These studies suggest that the activation in NLRP1 inflammasome and its signaling pathways is correlated to the pathogenesis of AD and maybe serve as therapeutic targets for AD.

Recent studies suggest that NLRP1 is highly expressed in females in some pathological states such as nodular melanoma, and may be related to female high-risk diseases (Verma et al., 2012; Wu et al., 2016). However, whether NLRP1 and its downstream signals are expressed with a gender difference in AD has not been elucidated. Thus, we explored the expression of NLRP1 signals in AD using APP/PS1^+/−^ mice of different sexes. Our data showed that NLRP1, Caspase 1 and IL-1β were much higher in the cortex of female APP/PS1^+/−^ mice than males. These data suggest that the differences in NLRP1 signals in the female and male APP/PS1+/- mice may be one of the reasons for the gender difference of AD.

Consistent with previous studies, our results also showed that Aβ plaque formation and neuronal apoptosis were significantly higher in the female APP/PS1+/- mice than males. However, whether the gender difference of NLRP1 signals in the brains of APP/PS1^+/−^ mice directly affects Aβ plaque formation and neuronal apoptosis, and participates in the development of AD was not completely elucidated in the present study, which requires further in-depth studies. However, the different brain metabolic profile in male and female mice given the different responds to systemic inflammation in the APP/PS1^+/−^ mice (Agostini et al., 2020), which may result in sexual dimorphism in inflammosome activation. In any case, our present data revealed that NLRP1 signals were pathologically overexpressed in CNS of female AD mice, which would at least provide a new insight to recognize the mechanisms of gender difference in AD.

## Data Availability Statement

The datasets generated for this study are available on request to the corresponding author.

## Ethics Statement

The animal study was reviewed and approved by The Experimental Animal Ethics Committee of Xinxiang Medical University.

## Author Contributions

XianW, WS, and JZ designed the experiments. JZ, LP, DZ, YX, XiaoW, YC, JL, and CC performed the experiments. JY analyzed the data. QG and WD raised the animal and collected the tissues. WS revised the manuscript. All authors contributed to the article and approved the submitted version.

## Conflict of Interest

The authors declare that the research was conducted in the absence of any commercial or financial relationships that could be construed as a potential conflict of interest.
